# Supplementary material for: Animal source food consumption practice and factors associated among infant and young children from selected rural districts in Ethiopia: A cross-sectional study
Source: PLoS One. 2024 Jul 5;19(7):e0306648. doi: 10.1371/journal.pone.0306648 (PMC11226061; doi:10.1371/journal.pone.0306648)
Supplement: S1 Table — (DOCX) [file pone.0306648.s001.docx]

Table 4 Bivariate Logistic regression on dairy consumption of infant and young children

| **Characteristics** | **Categories** | **COR(95%CI)** | **p.** |
| --- | --- | --- | --- |
| Milk Source Summary | Family Milk Cow/Goat | Ref |  |
|  | Local Market | 2.37(1.67, 3.37) | 0.000 |
|  | Others | 1.90(0.85, 4.26) | 0.121 |
| Roots Crop Production | No | 0.66(0.48, 0.92) | 0.014 |
|  | Yes | Ref |  |
| Fruits Production | No | 1.22(0.88, 1.68) | 0.24 |
|  | Yes | Ref |  |
| Cash Crops Production | No | 1.54(1.09, 2.16) | 0.013 |
|  | Yes | Ref |  |
| Household Food Security | Insecure | 2.14(1.54, 2.97) | 0.000 |
|  | Secure | Ref |  |
| MDD | ≤3 | 9.69(5.49, 17.11) | 0.000 |
|  | ≥4 | Ref |  |
| Ox Ownership | No | 0.54(0.37, 0.78) | 0.001 |
|  | Yes | Ref |  |
| Cow Ownership | No | 2.62(1.88, 3.66) | 0.000 |
|  | Yes | Ref |  |
| Goat Ownership | No | 2.31(1.39, 3.85) | 0.001 |
|  | Yes | Ref |  |
| Donkey Ownership | No | 2.61(1.62, 4.20) | 0.000 |
|  | Yes | Ref |  |
| Chicken Ownership | No | 2.24(1.61, 3.12) | 0.000 |
|  | Yes | Ref |  |
| Crop Production Diversity Practice | No | 1.42(0.99, 2.04) | 0.058 |
|  | Yes | Ref |  |
| Child Age in Months | 6-8 | Ref |  |
|  | 9-11 | 0.56(0.33, 0.95) | 0.03 |
|  | 12-23 | 0.67(0.44, 1.03) | 0.66 |
| Educational Status of the Mother | No education | 3.40(1.94, 5.97) | 0.000 |
|  | Grades 1-5 | 4.04(2.36, 6.91) | 0.000 |
|  | Grades 6-8 | 1.85(1.14, 3.00) | 0.013 |
|  | Grade 9 or Above | Ref |  |
| Estimated Annual income of the Household | <10000 | 2.47(1.36, 4.49) | 0.003 |
|  | 10000-20000 | 1.66(0.84, 3.26) | 0.014 |
|  | 20001-30000 | 1.04(0.47, 2.30) | 0.931 |
|  | >30000 | Ref |  |
| Maternal Occupation | Housewife | Ref |  |
|  | Merchant | 0.61(0.34, 1.10) | 0.102 |
|  | Farmer | 0.50(0.27, 0.91) | 0.023 |
|  | Other | 0.49(0.23, 1.07) | 0.075 |
| Agriculture Land in Hectare | <0.5 Hectare | 1.40(1.00, 1.95) | 0.045 |
|  | >0.5Hectare | Ref |  |
