# Supplementary material for: Animal source food consumption practice and factors associated among infant and young children from selected rural districts in Ethiopia: A cross-sectional study
Source: PLoS One. 2024 Jul 5;19(7):e0306648. doi: 10.1371/journal.pone.0306648 (PMC11226061; doi:10.1371/journal.pone.0306648)
Supplement: S2 Table — (DOCX) [file pone.0306648.s002.docx]

Table 5 Logistic regression analysis on Flesh/Meat Consumption of infant and young children

| **Characteristics** | **Categories** | **COR(95%CI)** | **p.** |
| --- | --- | --- | --- |
| Cereal Production | No | 4.04(0.51, 31.82) | 0.185 |
|  | Yes | Ref |  |
| Sex of the child | Female | 0.10(0.1, 0.76) | 0.026 |
|  | Male | Ref |  |
| Household food security | Insecure | 5.08(1.09, 23.71) | 0.039 |
|  | Secure | Ref |  |
| MDD | ≤3 | 26.95(5.72, 126.87) | 0.000 |
|  | ≥4 | Ref |  |
| Ox Ownership | No | 2.73(0.82, 9.07) | 0.102 |
|  | Yes | Ref |  |
| Cow Ownership | No | 2.41(0.70, 8.32) | 0.165 |
|  | Yes | Ref |  |
| Donkey Ownership | No | 3.73(1.07, 13.02) | 0.039 |
|  | Yes | Ref |  |
| Mothers’ Age | ≤26 | Ref |  |
|  | ≥27 | 3.05(0.65, 14.24) | 0.156 |
| Family Size | ≤5 | Ref |  |
|  | ≥6 | 3.12(0.68, 14.55) | 0.148 |
| Agricultural Land in Hectare | <=0.5Hr | 0.44(0.12, 1.52) | 0.193 |
|  | >0.5Hr | Ref |  |
