# Supplementary material for: Animal source food consumption practice and factors associated among infant and young children from selected rural districts in Ethiopia: A cross-sectional study
Source: PLoS One. 2024 Jul 5;19(7):e0306648. doi: 10.1371/journal.pone.0306648 (PMC11226061; doi:10.1371/journal.pone.0306648)
Supplement: S3 Table — (DOCX) [file pone.0306648.s003.docx]

Table 6 Bivariate logistic regression analysis on eggs consumption of infant and young children

| **Characteristics** | **Categories** | **COR(95%CI)** | **p.** |
| --- | --- | --- | --- |
| Root crops Production | No | 2.20(1.42, 3.41) | 0.000 |
|  | Yes | Ref |  |
| Legume Crops Production practice | No | 1.48(0.96, 2.28) | 0.078 |
|  | Yes | Ref |  |
| Vegetable Production | No | 1.34(0.86, 2.09) | 0.194 |
|  | Yes | Ref |  |
| Fruits Production Practice | No | 2.95(1.39, 3.47) | 0.001 |
|  | Yes | Ref |  |
| Cash Crops Production Practice | No | 1.38(0.86, 2.21) | 0.187 |
|  | Yes | Ref |  |
| Household Food Security | No | 2.56(1.63, 4.03) | 0.000 |
|  | Yes | Ref |  |
| MDD | ≤3 | 10.04(6.10, 16.54) | 0.000 |
|  | ≥4 | Ref |  |
| Ox Ownership | No | 1.99(1.25(3.17) | 0.004 |
|  | Yes | Ref |  |
| Cow Ownership | No | 1.71(1.11, 2.63) | 0.016 |
|  | Yes | Ref |  |
| Sheep Ownership | No | 1.93(1.04, 3.57) | 0.036 |
|  | Yes | Ref |  |
| Chicken Ownership | No | 4.17(2.62, 6.64) | 0.000 |
|  | Yes | Ref |  |
| Mothers’ Age | ≤26 | Ref |  |
|  | ≥27 | 1.57(0.99, 2.49) | 0.055 |
| Educational Status of the Mother | No Education | 2.99(1.23, 7.26) | 0.016 |
|  | Grade 1-5 | 1.59(0.78, 3.23) | 0.203 |
|  | Grade 6-8 | 0.69(0.38, 1.26) | 0.227 |
|  | Grade 9 and Above | Ref |  |
| Estimated Annual income of the Household | <10000 | 2.55(1.27, 5.13) | 0.009 |
|  | 10000-20000 | 1.03(0.48, 2.21) | 0.942 |
|  | 20001-30000 | 1.37(0.54, 3.51) | 0.511 |
|  | >30000 | Ref |  |
| Agricultural Land Size in Hectare | ≤0.5Hr | 1.79(1.13, 2.82) | 0.013 |
|  | >0.5Hr | Ref |  |
