# Supplementary material for: Animal source food consumption practice and factors associated among infant and young children from selected rural districts in Ethiopia: A cross-sectional study
Source: PLoS One. 2024 Jul 5;19(7):e0306648. doi: 10.1371/journal.pone.0306648 (PMC11226061; doi:10.1371/journal.pone.0306648)
Supplement: S4 Table — (DOCX) [file pone.0306648.s004.docx]

Table 7 Bivariate Logistic regression on overall ASF consumption of infant and young children

| **Characteristics** | **Categories** | **COR(95%CI)** | **p.** |
| --- | --- | --- | --- |
| Root Crops Production Practice | No | 1.75(1.26, 2.43) | 0.001 |
|  | Yes | Ref |  |
| Cash Crops Production Practice | No | 0.80(0.57, 1.12) | 0.194 |
|  | Yes | Ref |  |
| Mother got sick in the last two weeks | No | Ref |  |
|  | Yes | 1.55(1.11, 2.18) | 0.011 |
| Household Food Insecurity | Insecure | 2.50(1.80, 3.47) | 0.000 |
|  | Secure | Ref |  |
| MDD | ≤3 | 22.07(9.47, 51.42) | 0.000 |
|  | ≥4 | Ref |  |
| Ox Ownership | No | 1.91(1.30, 2.79) | 0.001 |
|  | Yes | Ref |  |
| Cow Ownership | No | 2.70(1.94, 3.76) | 0.000 |
|  | Yes | Ref |  |
| Goat Ownership | No | 2.36(1.39, 4.00) | 0.001 |
|  | Yes | Ref |  |
| Sheep Ownership | No | 2.10(1.22, 3.63) | 0.008 |
|  | Yes | Ref |  |
| Donkey Ownership | No | 2.51(1.54, 4.11) | 0.000 |
|  | Yes | Ref |  |
| Chicken Ownership | No | 2.88(2.06, 4.02) | 0.000 |
|  | Yes | Ref |  |
| Educational Status of the Mother | No Education | 3.84(2.18, 6.79) | 0.000 |
|  | Grades 1-5 | 3.75(2.19, 6.42) | 0.000 |
|  | Grades 6-8 | 1.69(1.02, 2.78) | 0.040 |
|  | Grade 9 or Above | Ref |  |
| Estimated Annual income of the Household | <10000 | 3.34(1.76, 6.31) | 0.000 |
|  | 10000-20000 | 1.62(0.79, 3.30) | 0.188 |
|  | 20001-30000 | 1.61(0.70, 3.69) | 0.262 |
|  | >30000 | Ref |  |
| Maternal Occupation | Housewife | Ref |  |
|  | Merchant | 0.65(0.36, 1.16) | 0.144 |
|  | Farmer | 0.47(0.25, 0.87) | 0.016 |
|  | Other | 0.48(0.22, 1.08) | 0.076 |
| Income Use Decision | Both | Ref |  |
|  | Wife | 1.75(1.12, 2.74) | 0.015 |
|  | Husband | 1.04(0.69, 1.56) | 0.856 |
|  | Other | 1.73(0.41, 7.35) | 0.457 |
| Agricultural Land Size in Hectare | ≤0.5Hr | 1.54(1.11, 2.12) | 0.009 |
|  | >0.5Hr | Ref |  |
| Head of the Household | Husband | Ref |  |
|  | Wife | 2.79(1.09, 7.19) | 0.033 |
|  | Other | 2.17(0.74, 6.32) | 0.156 |
