# Supplementary material for: Animal source food consumption practice and factors associated among infant and young children from selected rural districts in Ethiopia: A cross-sectional study
Source: PLoS One. 2024 Jul 5;19(7):e0306648. doi: 10.1371/journal.pone.0306648 (PMC11226061; doi:10.1371/journal.pone.0306648)
Supplement: Attachment — Submitted filename: Response to Reviewers.pdf [file pone.0306648.s007.pdf]

June 1, 2022

Subject: Point-by-point response to Editors guidance and reviewers' comments

Dear Editor,

Thank you for giving us the opportunity to submit a revised draft of the manuscript "Animal source food consumption practice and factors associated among infant and young children from selected rural districts in Ethiopia: A cross-sectional study" for publication in PLOSOne. We would like to thank the reviewers for their precious time and constructive comments. We have addressed the comments in a point- by- point response and a revised manuscript. The changes are highlighted in with track changes in the revised manuscript.

Journal Requirements:

When submitting your revision, we need you to address these additional requirements.

1. Please ensure that your manuscript meets PLOS ONE's style requirements, including those for file naming. The PLOS ONE style templates can be found at

https://journals.plos.org/plosone/s/file?id=wjVg/PLOSOne_formatting_sample_main_body.pdf and https://journals.plos.org/plosone/s/file?id=ba62/PLOSOne_formatting_sample_title_authors_affiliations.pdf

- **Response**: Thanks for the comment, and sharing link for formats and guidance. Changes are made accordingly.

2. In the ethics statement in the Methods, you have specified that verbal consent was obtained. Please provide additional details regarding how this consent was documented and witnessed, and state whether this was approved by the IRB.

- **Response:** Thanks for the feedback. Detailed Ethics statement is added to the methods and material subsection.

3. We note that you have indicated that data from this study are available upon request. PLOS only allows data to be available upon request if there are legal or ethical restrictions on sharing data publicly. For more information on unacceptable data access restrictions, please see http://journals.plos.org/plosone/s/data-availability#loc-unacceptable-data-access-restrictions.

In your revised cover letter, please address the following prompts:

a) If there are ethical or legal restrictions on sharing a de-identified data set, please explain them in detail (e.g., data contain potentially sensitive information, data are owned by a third-party organization, etc.) and who has imposed them (e.g., an ethics committee). Please also provide contact information for a data access committee, ethics committee, or other institutional body to which data requests may be sent.

b) If there are no restrictions, please upload the minimal anonymized data set necessary to replicate your study findings as either Supporting Information files or to a stable, public repository and provide us with the relevant URLs, DOIs, or accession numbers. For a list of acceptable repositories, please see http://journals.plos.org/plosone/s/data-availability#loc-recommended-repositories.

We will update your Data Availability statement on your behalf to reflect the information you provide.

- **Response:** Data uploaded.

4. Please include your full ethics statement in the 'Methods' section of your manuscript file. In your statement, please include the full name of the IRB or ethics committee who approved or waived your study, as well as whether or not you obtained informed written or verbal consent. If consent was waived for your study, please include this information in your statement as well.

- **Response**: Detailed ethics statement is included in the methods section.

Reviewer #1:

- Why do you prefer single proportion formula while you are assessing the predictors/levels of significance/
    - **Response:** Thanks for picking the point. Yes, it would have been good if sample size was calculated or the second section of the objective, predictor identification. However, to the level of literature search conducted before implementation of the current research there was no study that aimed to identify predictors of animal source food consumption among infant and young children. Thus, we were obliged to do the research using sample size computed with the application of single proportion formula. Luckily, the absence of literature on factors contributing to the inclusion of animal source foods in diets/complementary foods of infant and young children make this study a value adding article.

- The reason for selection of three dstrict (Why purposive)? can we generate an evidence on the selected study population to the general remaining population?

- **Response:** Sure this is also an interesting pick! The districts were selected because they were areas where a project that aimed to link cattle nutrition with human nutrition was being implemented. They are selected purposively for this study, but randomly for the broader project that funded this analysis. Sure, we can generate inferable evidence as the districts were selected randomly by the broader project though this study used them purposively as it is part of the wider Nutrition Sensitive Agricultural project.

- About Ethical approval of two different regional administration? Who should provide Ethical approval? IRB or National Research Ethical Review Committee? Nothing displayed (Reference number??)
Does your research Ethical approval from Oromia or Sidama?

  - **Response**: Good point! Description about research ethics was included in the online declaration assuming it will be included in the publication following acceptance of the manuscript. The research was reviewed and approved by Institutional Review Board of Hawassa University. Descriptions are included in the method subsection of the manuscript.

Reviewer #2:

It is an important research topic but the quality of the reporting and validity and reproducibility of methods employed to address the stated problem is unsatisfactory. The results and discussion sections need more improvement. Moreover, grammatical and editorial errors are frequent thought the document and thus the manuscript needs major revision.

- **Response**: Thanks for echoing that the research topic is important. And that is why we tried to address it with all the limitation. Concerning validity and reproducibility of the methods employed we believe that it is to the level of epidemiological recommendations and nutritional assessment guidance. As no method and procedure to be used for research is free of drawbacks, the current research shall also shoulder this universal existence of limitations in methods and procedures to be applied for research execution. With all these, the study employed cross-sectional study design and followed standard sampling procedures that are described in the manuscript. Data was managed with acceptable statistical analyses and results were interpreted to the level best. Tool to collect data was compiled from recommended standard tools and literature review. Results and discussion section are revised. Grammatical and editorial errors are considered with the help of language experts. Overall, we accepted the recommended need for major revision and made it effective.

Specific Comments

- **Title:** The title needs modification in light of the cross-sectional nature of the study design, authors can't determine predictors of animal Sources Food Consumption among Infants and Young Children using a cross-sectional study design. For determining predictors authors need to use either a cohort or an interventional study design. By using a cross-sectional study, one can determine only the associated factors for the outcome variable. Moreover, the phrase "**Rural Districts of Ethiopia" is very broad considering the large surface area of Ethiopia.**
    - **Response:** Corrections are made as per the recommendation. To narrow the scope of the title in terms of geographic coverage included the word "selected."

Abstract:

- In the background section of the abstract, the problem statement and rationale for conducting the study are missing. There is a miss-match between the title and the specific objectives described in the abstract. In the title, "Determination of predictors …" while in the objective "assess consumption of ASF and identify their predictors".
    - ✓ **Response**: Good point! Yes it was lacking just to shorten the abstract as the information is well addresses in the background. We accepted the comment and included the research gap. Revision was made to manage the observed mismatch between title and objective.
- In the method section: Authors failed to describe the specific study area, the sampling procedure, the type of data collected, who collected the data, and what type of descriptive analysis was carried out.
    - ✓ **Response:** Recommendation is accepted and information included.
- Results Section: the authors used logistic regression analysis to identify associated factors with animal source food consumption. However, they use p values to report results. They should use AOR along with 95% CI as this help us to report the statistical significance, strength of association and direction of association.
    - ✓ **Response:** All right. It would have been good if AOR with 95%CI are included. But we only used p values to make the abstract brief and informative. Comment accepted and AOR with 95%CI included.

Introduction: Authors need to explicitly describe the problem statement for their study.

- ✓ **Response:** Thanks for the comment and recommendation. Revision is made to explicitly indicate problem statement. At the end of the Introduction section of the manuscript it was described as "Despite the less inclusion if ASF in diets of IYC, pertaining to researches' focus, assessment on the consumption of ASF and

identifying contributing factors were less addressed as a primary outcome of the studies."

Methods:

- The study areas were not adequately described including population distribution, geography, socio-economy status and health infrastructure etc.
    - ✓ **Response**: Comment accepted and study area description broadened.
- On Page 4, 2nd paragraph, the authors used "Sampling" as a title for Sample Size Determination and Sampling Procedure. The word sampling is incomplete and thus should be replaced by Sample Size Determination and Sampling Procedure.
    - ✓ **Response**: recommendation accepted and correction made!
- In sample size calculation, authors didn't provide the expected prevalence assumed for calculation of the sample size and didn't consider for non-response.
    - ✓ **Response**: Comment accepted and process followed described.
- In the sampling procedure, authors said that they selected randomly four Kebelle from each district but they didn't elaborate why they selected four Kebelles as number of Kebelles can vary from district to district. They didn't provide also justification why they selected 25 study subjects from each Kebelle. There is no "Equal Proportional Allocation" sampling technique. It is not also clear how the systematic sampling method was conducted.
    - ✓ **Response**: Thanks for the observation and comment. Sure, the study is done as an extension of a developmental Nutrition Sensitive Agricultural intervention project, and aligning all academic requirements of conducting a research with such development works used to be challenging. However, we believe that we tried to address basics of sampling with all must to shoulder difficulties and also described the methodological recommendations in the discussion section of the manuscript as recommended.
- On Page 4, 3rd paragraph, First sentence, Authors wrote that "*Data were collected through one-on-one interviewer administered questionnaire*". What does "One-on-one" mean? Is it meant to say face-to-face?
    - ✓ **Response:** Good to pick such confusing narrations. It was to mean no mass data collection was made. By saying "One-on-one", we meant each participant was treated separately for data collection. It is also an explanation for privacy related concerns. Comment is accepted and changed in to face-to-face.
- Authors didn't describe how they measured the independent and dependent variables. No operational definition was given. No information was given as to who collected the data.
    - ✓ **Response**: In the "Data Collection" sub section of the manuscript we have described how and using what primary outcome (Animal Source Food

Consumption practice) was measured and appropriate citation are made. Accepted concern related with information on data collectors and included a sentence.

- The data quality control used was inadequate. Did authors translate their tool to local language? Did they use supervision during data collection? Did they check completeness of the collected data on daily basis?
    - ✓ **Response**: Thanks for the detailed review, comment and recommendation. All the points raised were conducted during implementation of the study but missed in the narration. Corrections are made accordingly.
- With regards to ethical consideration, authors claimed that "*All methods were carried out in accordance with the Declaration of Helsinki*". This is vague, incomplete and inappropriate description. This should not be described under the "Data collection" heading. There is no information as to which University Institutional Review Board gave the permission? Dis authors use verbal or written informed consent? What did they do to ensure autonomy, beneficence and do not harm?
    - ✓ **Response:** Ethics statement added to the methods section.
- In the data analysis section, authors failed to include multicollinearity test and goodness of fit test for the final logistic model.
    - ✓ **Response**: Thanks for pointing out non reported assumption checks. Included.

Results:

- What is the response rate for the study? It needs to be stated.
    - ✓ **Response**: Included.
- On page 6, Table 1: Second column should be labeled as categories; in Table 1 and Table 2, the column label for the third and fourth column should be revised as Frequency (N) and Percentage (%), respectively. What is "Age completed years" mean in Table 1? It is not clear and why it is categorized as $\leq$ 26 and $\geq$ 27.
    - ✓ **Response:** Frequency and percentage labeling made. Age in completed years is to mean that we have not considered months and not rounded for it. Changed in to the common "Age." Concerning age categorization we use mean score (25.6Yrs) for age of our participants.
- On Page 8, Table 2: The reason for categorizing family size, crop production and livestock ownership is not clear. Family size is categorized into $\leq$5 and $\leq$6. In the crop production and livestock ownership, what if a farmer produces two, three, four, five or all type of crops and a farmer owns two or more of the livestock?
    - ✓ **Response**: family size categorization was in consideration of observed median score and average Ethiopian family size. The "$\leq$" sign before 6 is a typo error and replaced with "$\geq$." Concerning categorization of crop production, data was collected about what crop/s a household used to produce and grouped in to the categories (Cereal, Legumes, Vegetable….) assuming nutrition classification. A Household was counted for more than one groups of crop if produced crops to be

grouped in different crop categorization. That is why the results become more than the total number of participants. Results are reported only for producers. For example, in the result it is reported that n=433/71.6% of households produce cereals (maize, barely, sorghum, wheat…..). This means the remaining households (n=173) do not produce cereals. The report about livestock only ownership. Though data on numbers owned was collected there is no much difference in number of ownership. Thus, we dropped number and reported only ownership.

- On Page 9: The y-axis of figure is not labeled
  - ✓ **Response**: It is labeled.
- Page 11, Table 3: The outcome variable (animal source food consumption) frequency and percentage is missing in Table 3. It doesn't include also the bivariate analysis results (COR along with the 95% CI). Why is donkey ownership included in Table 3 while Table is about animal source food consumption? How ownership of donkey is can be associated with the consumption of animal source foods? The bivariate and multivariable results should be presented in the same Table. It is not also clear why some variables and categories are missed in Table 3.
  - ✓ **Response:** Yes, it would have been good if frequency and percentage of animal source food consumption and bivariate analysis results (COR along with the 95%CI) are included in Table 3. However, the congestion they create to the table outweighs than the value they add to it. We imagined the number columns need to be added to the table and decided not to include. For the sake of helping readers interest with the results we have we submitted bivariate results as supporting file. Yes, donkey is not a direct source of animal source food. But can contribute to the consumption in different pathway like income generation, transportation and others. The assumption or logic behind including donkey in the model is similar with the other non-direct source independent variables. The possible reasoning for the observed association is addressed in the discussion section. Yes some variables and categories are missing from Table 3. Variables are missing from the table because the analysis we did is forward stepwise logistic regression and we did not include eligible for multivariable but non-associated variable. It is to make the Table to the point and as simple as possible for reader. Reference categories are removed from the Table as they are well pointed and included in the text interpretation of results.

Discussion:

- Generally the discussion section is very brief considering the study objectives and the findings.
  - ✓ **Response**: Thanks for the comment. Yes it looks brief but considered available literature and central interest of the research. Possibly because of non-availability

of comparable publication to exhaustively discuss finding on factors associated. Rather than as limitation of the current research, this shall be considered as the value this research work is adding to the existing body of knowledge and literature.

- On page 12, 1st paragraph, comparison of proportion from various areas was based narrative descriptions. It needs to be supported by data/figures. For example in the sentence "*The proportion of IYC who consumed flesh is comparable with reports from Aleta Wondo [25], Gorche [12], Dangila [11], Wollo [26] districts in Ethiopia, but lower than proportions from Addis Ababa city [27], Afar [28], Bale [29] and Wolaita Sodo [30] towns in Ethiopia*" what was the figure for Aleta Wondo? Gorche? Dangila? Wollo? etc. to say greater than or lower than the current study. Moreover, implications of the findings to clinical/public health practices need to be described.
    - ✓ **Responses:** statistical figures from the cited articles are included and implications are pointed.
- On page 12, 2nd paragraph, associated factors ("Predictors") with the consumption of animal source food are discussed. However, the results are not compared with other findings. Simiarly, implications of the findings are not discussed.
    - ✓ **Response:** Thanks for the logical and reasonable observation. Yes results on predictors or factors associated with animal source food consumption are not compared with other findings. As mentioned above for comments on sample size calculation, we failed to do so because articles to be used for the purpose are lacking. We appreciate if this also considered as the manuscript we submitted touched untouched segment of infant and young child nutrition.
- Limitations of the study related to selection and measurement biases and confounding are not discussed.
    - ✓ **Response**: Thanks for interesting critics. We did not mention selection and measurement biases as they are described in the methods and materials section. Concerning confounding, as it can be controlled during analysis we believe that the forward stepwise multivariable analysis addressed it. Moreover, accepting the comment for inclusion of limitations clearly and directly in the discussion section, we have described them.

With Best regards,

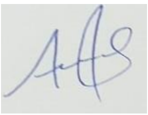

Alemneh Kabeta Daba (PhD)
