# Supplementary material for: Animal source food consumption practice and factors associated among infant and young children from selected rural districts in Ethiopia: A cross-sectional study
Source: PLoS One. 2024 Jul 5;19(7):e0306648. doi: 10.1371/journal.pone.0306648 (PMC11226061; doi:10.1371/journal.pone.0306648)
Supplement: Attachment — Submitted filename: PONE-D-22-30710_R1_reviewer_comments.pdf [file pone.0306648.s008.pdf]

Manuscript Click here to access/download;Manuscript;Manuscript.docx

Animal source food consumption practice and factors associated among infant and young children from selected rural districts in Ethiopia: A cross-sectional study

Alemneh Kabeta Daba[1*], Mary Murimi[2], Kebede Abegaz[3], Dejene Hailu[4]

[1]School of Nursing, College of Medicine and Health Sciences, Hawassa University, Hawassa, Ethiopia
[2]Department of Nutritional Sciences, College of Human Sciences, Texas Tech University, Lubbock, Texas, USA
[3]School of Nutrition and Food Science and Technology, College of Agriculture, Hawassa University, Hawassa, Ethiopia
[4]School of Public Health, College of Medicine and Health Sciences, Hawassa University, Hawassa, Ethiopia
[*]Corresponding Author: alemnehkabeta@hu.edu.et (AKD)

Abstract

Background: Children are recommended to consume animal source foods (ASF) as part of diversified diets. However, children's ASF consumption practice is less studied and contributing factors are not exhaustively identified. In view of that, the purpose of this study was to assess consumption of ASF and identify associated factors among 6-23 months old children from selected rural districts in Ethiopia.

Methods: A cross-sectional study was conducted on a total of 606 6-23 months old children from selected rural districts of Oromia and Sidama regional states in Ethiopia. A two-stage sampling technique was used to select participants. Data were collected using interviewer-administered questionnaire. Frequencies, percentages and mean scores with standard deviations were generated to describe the participants and report univariate outcomes. Consumption of ASF was assessed using a 2[illegible]urs dietary recall. Logistic regression analysis was applied to identify contributing factors to ASF consumption.

Results: Household food security increased the likelihood of dairy [AOR=1.66(95%CI: 1.16 2.38), P =0.006], eggs [AOR=2.15 (95%CI: 1.33, 3.49), P =0.002] and meat [AOR=5.08 (95%CI: 1.09, 23.71), p =0.039] consumption by the children. Cow [AOR=1.86(95%CI: 1.28, 2.70), P =0.001], donkey [AOR=1.83(95%CI: 1.08, 3.11), P =0.024] and chicken [AOR=1.53 (95%CI: 1.05, 2.22), P =0.027] ownership increased the odds of dairy con[illegible]ption. Grades five up to eight [AOR=1.74 (95%CI: 1.06, 2.86), P =0.028] or grades 9 or above [AOR=2.96 (95%CI: 1.62, 5.42), P <0.001] maternal educational achievements wer[illegible]o associated with better dairy consumption. Children from households that owned chicken, [AOR=3.20 (95%CI: 1.97, 5.19), P <0.001] and produce root crops [AOR=1.67 (95%CI: 1.05, 2.66), P =0.031] were more likely to consume eggs than their counters.

Conclusions: Low proportion of children consumed ASF. Household food security, livestock ownership, household income, root crop production and maternal education contributed to ASF consumption. Nutrition sensitive agricultural extension activities aided by nutrition education should be considered and evaluated for their effect on children's ASF consumption practice.

Keywords: Animal Source Food, Consumption, Infant and Young Children, Rural, Ethiopia

Introduction

The first two years of life are critical period during which organs, body systems and immunity develop and children start to learn the environment [1]. During this period, good nutrition-supported by appropriate behavioral interventions plays an important role to help children grow and develop to their full potential and have he[illegible]er foundation for the rest of their lives [2]. The period is long portion of the first 1000 days, which [illegible]onsidered as a window of opportunity during which good nutrition practice has to continue and corrective nutrition interventions has to be practiced to rectify nutritional derangements t[illegible] happened during pregnancy [3]. If not, the wrong face of the period takeover to change it to a window of vulnerability. A[illegible]result, for children who used not meet the quantity and quality of nutrition their body require, the period was found being a season in which they lose much of their growth and developmental potential [4].

Growing[illegible]ldren are fully dependent on mature individuals around them for general care, nutrition and interaction. To support nutrition literacy of the care providers in order to serve children to meet their nutritional requirement, infant and young child feeding (IYCF) guides [5–8] have been developed. The guides mainly focus on breast and complementary feeding practices during the first two years of life. As one of the complementary feeding recommendations, dietary diversity is a useful indicator of nutritional quality of complementary foods consumed by infants and young children (IYC) [9]. To satisfy the minimum dietary diversity [illegible]DD) recommendation, IYC are expected to consume at least four of the seven food groups (cereals and roots, legumes, vitamin A rich fruits and vegetables, other fru[illegible]nd vegetables, dairy products, eggs and flesh) in a day. Animal source foods share 43% (three of the seven food groups) of the food groups used to determine whether IYC met or not [illegible]). But, more than 70% of IYC in Ethiopia did not [illegible]MDD [10–13], and ASF [illegible]e consistently reported as the commonly missed food group[illegible]espite the less inclusion if ASF in diets of IYC, pertaining to researches' focus [illegible]essment on the consumption of ASF and identifying contributing factors were less addressed as a primary outcome of the studies. [illegible]ordingly, the purposes of this study were to assess consumption of ASF practice[illegible]d identify associated factors among IYC from selected rural districts in Ethiopia. The findings have potential to helping the realization of

Ethiopian food and nutrition policy and strategy through food based approach that aimed at tackling malnutrition in all its forms.

Materials and methods

Study area and design

The study was conducted in Sidama and Oromia regional states of Ethiopia. Three districts (Arsi Negelle, Wondo Gent and Dale) were purposively selected from the two regional states as they were implementation areas for a project that ain[illegible]o link cattle nutrition with human nutrition. Arsi Negele district/woreda is one of the districts in the Oromia Region of Ethiopia. Arsi Negele is bordered on the south by Shashemene Zuria, on the southwest by Lake Shala which separates it from Shala, on the west from the Sidama Region, on the north by Misraq Shewa with which it shares the shores of Lake Abijatta and Langano, and on the east by the Arsi Zone. The altitude of this woreda ranges from 1500 to 2300 meters above sea level. Data of the land in the woreda shows that 29.9% is arable or cultivable[illegible]% pasture, 5.2% forest, and the remaining 60.6% is considered swampy, degraded or otherwise unusabl[illegible]ased on figures published by [illegible] Central Statistical Agency of Ethiopia (200[illegible]his woreda has an estimated total population of 198,307, of whom 100,626 are men and 97,681 are women. About 21% (42,054) of its population are urban dwellers. The estimated area of the woreda is 1,400.16 square kilometers. The majority of the inhabitants were Muslim (68.86%)[illegible]lowed by Ethiopian Orthodox Christian (20.2%), Protestant (8.99%) and Catholic (1.04%). The four largest ethnic groups in Arsi Negele are the Oromo (85.92%), the Amhara (7.69%), the Kambata (2.73%), and the Sodo Gurag[illegible]08%); all other ethnic groups made up 2.58% of the population. Oromiffa was spoken as a first language by 83.65%, 11.89% spoke Amharic, and 2.44% spoke Kambata; the remaining 2.02% spoke [illegible] other primary languages reported. There were 33 Farmers Associations with 21,777 members and 12 Farmers Service Cooperatives with 11,430 members (CSA, 2007a[illegible]bout 22% of the total population has access to drinking water.

Dale and Wondo Genet districts are from [illegible]ama regional states of Ethiopia. Wondo Genet Woreda is one of the woreda[illegible]the Sidama Region of Ethiopia. It is bordered on the south by Malga, on the west by Awash Zuria, and on the north and eas[illegible]the Oromia Region. Based on the census conducted by the Central statistical Agency of Ethiopia, the woreda has a total population of 155,715, of whom 79,664 are men and 76,051 women. About 15% (23,125) of its population are urban dwellers. The majority of the inhabitants were Protestant (83.26%) followed by Muslim (7.4%), Ethiopian Orthodox Christian (6.69%) and Catholic (1.68%) (CSA, 2007b). Dale is the other woreda in the Sidama Region of Ethiopia. It is bordered on the south by Aleta Wendo and Chuko, on the west by Loka Abaya, on the northwest by Boricha, on the north by Shebedino, and on the east by Wensho. The elevation of this woreda varies from about 1200 meters above sea level along the shores of Lake Abaya to about 3200 meters at its western most 21 point. Report about land in Dale shows that 81.9% is arable or cultivable, 2.7% forest, and the remaining 15.5% is considered swampy, degraded or otherwise unusable. Important cash crops for Dale include corn, barley, haricot beans, local varieties of cabbage, and sweet potatoes.

Coffee is also an important cash crop in Dale, with 15.38 square kilometers planted with this crop. Based on the census conducted by the Central Statistical Agency of Ethiopia (200[illegible]e woreda has a total population of 242,658, of whom 122,918 are men and 119,740 women. The majority of the inhabitants were Protestants (79.98%) follow[illegible]y Ethiopian Orthodox Christian (8.04%), Muslim (4.69%), Catholic (3.46%) and tradition[illegible]gions (1.3%). The four largest ethnic groups in Dale were the Sidama (91.29%), the Amhara (3.98%), the Oromom (1.16%), and the Welayta (1.01%); all other ethnic groups made up 2.56% of the population. Only 22% of the inhabitants have access to drinking water [illegible]A, 2007b). A community-based cross-sectional study was applied from May to August 2018.

Sample size determination and sampling procedure

Sample size was calculated using single population proportion formula [14] at 95% confidence level, 0.05 margin of error and design effect of 1.5. Dairy consumption prevalence of 60.7% (12) was used to decide the sample size. Though a 5% non-response rate was considered during sample size determination, it was not applied for data collection as no non-responder was encountered. A two-stage sampling technique was used to select the enumeration kebeles and IYC. At stage-one four kebeles (n=12), the lowest unit in the administrative structure of Ethiopia, were selected randomly from each of the study districts (n=3) as enumeration areas. Stage-two also involved two activities. First, a house-to house census was conducted to develop a list of eligible households, households with IYC, for each kebele. Secondly, an equal proportional allocation was used to select households with IYC from each of the kebele. Accordingly, a total of 606 IYC (25 from each kebele) were selected through a systematic random sampling technique. The "K" value for each kebele was determined dividing the total number of eligible households by 25, number of IYC each kebele expected to contribute to the study sample size.

Data collection

Data were collected through face-to-face interviewer administered questionnaire. All the respondents were mothers of the IYC. The data collection questionnaire consisted sections for house[illegible] food in/security[15], dietary diversity assessment tool focused on ASF consumption practi[illegible]6] and socio-demographic and economic characteristics of the IYC, mothers and households. Household food in/security was assessed on a recall basis during thirty days before the date of data collection. Consumption of ASF was assessed as part of dietary diversity assessment over 24-hours period before the day of data collection. The tool was translated to local languages (Oromiffaa and Sidamu Afoo), tested and necessary amendments were made before it was used for [illegible] actual data collection. Nutrition and health professionals with a minimum of first degree collected the data. Data collectors attended three days training on basics of research methods, data collection techniques, research ethics and purpose of this study.

Completeness, quality and consistency of data were checked by the investigators, who served as field supervisors, at spot during the data collection at the field.

Data analysis

Data were checked for completeness, coded, entered into SPSS version 20 for windows and cleaned. Overall ASF consumption data were generated [illegible]ming up “0”-not consumed in the last 24-hours and “1”-consumed in the last 24-hours coding for flesh foods/meat, eggs and dairy consumption statuses. The summation results for overall ASF consumption ranged from zero to three. Zero resulted for IYC who consumed none of the ASF, 1 for IYC who consumed one of the ASF, 2 for IYC who consumed two of the ASF and 3 for IYC who consumed three of the ASF. Then, results of the summation for overall ASF consumption data were dichotomized in to consumed at least one of the ASF (“coded with 1”) and consumed none of the ASF (“coded with 0”) during the 24-hours before the survey.
Descriptive statistical outputs (frequencies, proportion and mean/median scores with SD/IQR) were computed to describe the participants and generate results on ASF consumption practice. Bivariate logistic regression analysis was done to identify eligible variables to be included in multivariable logistic regression model to identify predictors [illegible]SF consumption. Based on the results of bivariate logistic regression, variable with p[illegible]25 [17] were included in multivariable logistic regression model. Goodness of fit test was assume[illegible]or data distribution, and multicollinearity was checked using a variance inflation factor (VIF) more tha[illegible]. Multivariable forward stepwise logistic regression analysis was applied to identify factors associated with flesh foods, eggs, dairy and overall ASF consumption practice at statistical significance level of 0.05 or below.

Ethics statement

Letter of ethical clearance with ***Ref No: IRB/027/10*** was issued after the research proposal was reviewed by Institutional Review Board (IRB) of Hawassa University-College of Medicine and Health Sciences. Participation was on voluntary basis. Informed written consent and assent were obtained from mothers of the IYC.

Results

Maternal and children characteristics

Response rate of the study is 100%. An observation was dropped because of mixed response for ASF consumption practice. About two-thirds of the mothers attended school from grades one up to eight (64%), are Sidama (63%) by ethnicity and protestant (67.6%). Near to two-thirds (60%) of the mothers were ≤26 years old with a mean sco[illegible]D) of 25.6(5). Four out of five (79.5%) mothers were housemakers. Half (50.2%) of the children were male, while near to two-thirds (60.2%) were twelve to twenty three months old with a mean sc[illegible]SD) of 13.9 (5.4) (**Table 1**).

Table 1 Frequency and percentage distribution of maternal and children characteristics (n=605)

**Maternal Characteristics**	**Categories**	**Frequency (N)**	**Percentage (%)**
Education	No Formal Education	119	19.7
	Grades 1-4	159	26.3
	Grades 5-8	231	38.2
	Grade 9 and Above	96	15.9
Ethnicity	Sidama	381	63.0
	Oromo	187	30.9
	Wolayta	15	2.5
	Others	22	3.6
Religion	Protestant	409	67.6
	Muslim	164	27.1
	Orthodox	23	3.8
	Catholic	9	1.5
Age	≤26	363	60.0
	≥27	242	40.0
Occupation	House Wife	481	79.5
	Merchant	50	8.3
	Farmer	47	7.8
	Others	27	4.5
**Children Characteristics**			
Sex	Female	301	49.8
	Male	304	50.2
Age in months	6-11	241	39.8
	12-23	364	60.2

Household characteristics

Nearly all (93.6%) households were male[illegible]ded. Women were reported to decide on purchase of household daily need f[illegible]ore than half (58.2%) of the families. More than half of the households were with five or less family siz[illegible]ith a median score of 5 (IQR: 4, 7). Half of the households (53.4%) had two or more under-five children. The estimated annual income for 65.5% of the households was ≤10,000 Ethiopian Birr (ETB)/$312 (at $32[illegible]hange rate). Half (52.4%) of the households were food insecure. Local market (63%), family milk cow and/or goat (32.4%) and relatives and neighbors (4.6%) were reported as the usual source of milk for the households. More than half (56.2%) of the households owned more than half hectare of agricultural land. Cereal (71.6%), cash crops (66%), vegetables (56.7%), legumes (47.4%) and roots (40.3%) were the major crops produced by the households. Cows (42.5%), chicken (41.2%), oxen (23.8%) and donkeys (13.7%) were livestock owned by the households (**Table 2**).

Table 2 Frequency and percentage distributions of household characteristics (n=605)

Characteristics	Categories	Frequency (N)	Percentage (%)
Head of the Household	Husband	566	93.6
	Wife	23	3.8
	Other	16	2.6
Decision on Daily Household Needs made by;	Both Husband and Wife	186	30.7
	Wife	352	58.2
	Husband/Other	67	11.1
Family Size	≤5	360	59.5
	≥6	245	40.5
Number of Under-five Children	One	282	46.6
	Two and More	323	53.4
Estimated Annual income in Ethiopian Birr	≤10000/$312	396	65.5
	10001 – 20000/$312-624	110	18.2
	20001 – 30000/$624-937	49	8.1
	>30000/$937	50	8.3
Food Security	Food Insecure	317	52.4
	Food Secure	288	47.6
Agriculture Land Ownership	≤0.5 Hectare	265	43.8
	>0.5 Hectare	340	56.2
Crop Production: Produce-	Cereal	433	71.6
	Roots	244	40.3
	Legumes	287	47.4
	Vegetables	343	56.7
	Cash Crops	399	66
	Produce diversity of crops	429	70.9
Livestock Ownership	Oxen	144	23.8
	Cows	257	42.5
	Goats	69	11.4
	Sheep	62	10.2
	Donkey	83	13.7
	Chicken	249	41.2

Dairy products, eggs, flesh foods and overall ASF consumption practice of the IYC

Data on ASF consumption practice of the IYC was collected with the application of the standard dietary diversity assessment tool in which sever[illegible]d groups are listed including selected ASF (dairy products, eggs and flesh foods) [16[illegible]onsumption of dairy products, eggs, flesh foods was assessed for all of the IYC participated in this study over 24-hours[illegible]od prior to the data collection day. Because the primary interest of the current investigation is about the consumption practice of ASF by IYC, results are focused to [illegible]y products, eggs, flesh foods and overall ASF. Accordingly, dairy products were consumed by 41.2% of the IYC while 16.4% consumed eggs and 2.3% consumed flesh foods. Collectively, less than half (48.3%) of the children consumed at least one of the three ASF over 24-hours period prior to the data collection day **(Figure 1)**.

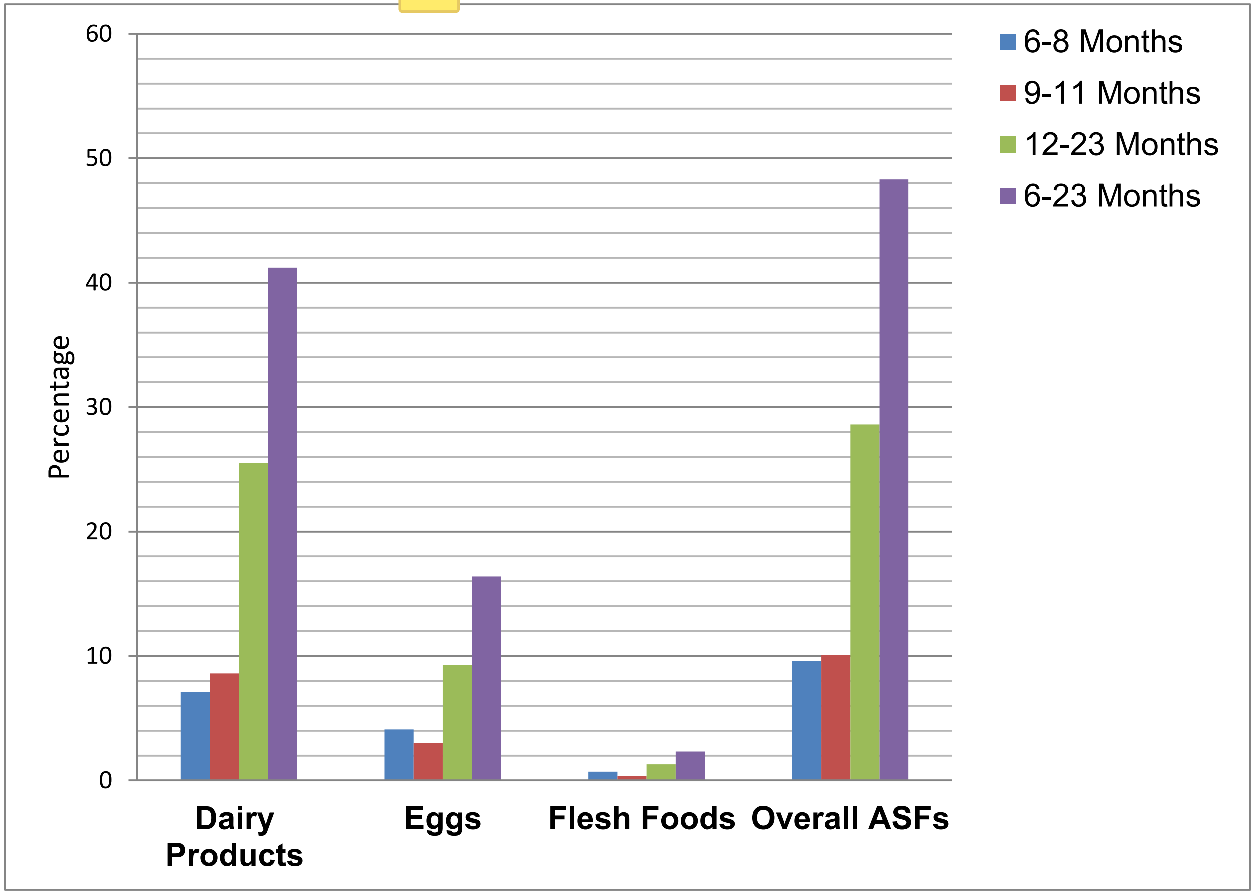

Figure 1 Percentage distributions of children who consumed selected ASF, according to WHO recommended age category

Factors associated with animal source foods consumption practice

Household food security was associated with specific and overall ASF consumption. Infant and young children lived in food secure households were [illegible] likely to consume dairy [AOR=1.66(95%CI: 1.16 2.38), p. =0.006], flesh/meat [AOR=5.08 (95%CI: 1.09, 23.71), p. =0.039], eggs [AOR=2.15 (95%CI: 1.33, 3.49), p. =0.002] and at least one of the ASF [AOR=1.94 (95%CI: 1.36, 2.78), p. <0.001] than IYC lived in food insecure households.

Educational status of the mothers and household level livestock ownership predicted dairy consumption. Infant and young children lived in househ[illegible] that owned cow [AOR=1.86([illegible]CI: 1.28, 2.70), p. =0.001] or chicken [AOR=1.5[illegible]%CI: 1.05, 2.22), p. =0.027] or donkey [AOR=1.83(95%CI: 1.08, 3.11), p. =0.024] were about two times more likely to consume dairy than IYC who lived in households th[illegible]d not owned cow, chicken [illegible]nkey. Infant and young children with mothers who achieved [illegible]es five up to eight educatio[illegible]e two times [AOR=1.74 (95%CI: 1.06, 2.86), p. =0.028] and IYC with mothers who achieved grade nine or above education were three times [AOR=2.96 (95%ci: 1.62, 5.42), P. <0.001] more likely to consume dairy than IYC with mothers who never attended formal education.

Root crops production and chicken ownership were associated with [illegible] consumption. Infant and young children from households that owned chicken were three times [AOR=3.20 (95%CI: 1.97, 5.19), p. <0.001] more likely [illegible]nsume eggs than IYC from households that did not own chicken. Infant and young children who resided in households that produces root crops were also about two times [AOR=1.67 (1.05, 2.66), p. 0.031] more likely [illegible]nsume eggs than children in households that did not produce root crops.

Livestock ownership, estimated annual income of the households and educational status of the mothers predicted [illegible]sumption of at least one of the ASF. Infant and young children from households that owned cow [AOR=1.89 (95%CI: 1.31, 2.74), p. =0.001] or chicken [AOR=1.93 (95%CI: 1.33, 2.81), p. =0.001] were two times more likely to consume at least one of the ASF than IYC from households that did not own cow or chicken. Infant and young children from households with an estimated annual income of 10,001-20,000 [AOR=1.70 (95%CI: 1.06, 2.73), p. =0.028], 20,001-30,000 [AOR=1.96 (95%CI: 1.01, 3.79), p. =0.047] and more than 30,000 [AOR=2.71 (95%CI: 1.36, 5.39), p. =0.005] were two to three times more likely to consume at least one of the ASF than IYC from households with an estimated annual income of 10,000ETB or less. Infant and young children with mothers who achieved grades five up to eight education were two times [AOR=2.03 (95%CI: 1.25, 3.31), p. =0.004] more likely to consume at least one of the ASF than IYC with mothers who never attended formal education (**Table 3**).

Table 3 Logistic regression analysis on factors associated with dairy products, flesh/meat, eggs and overall animal source foods consumption practice (n=605)

Characteristics	Categories	AOR(95%CI)	*p.*
**Dairy Products**			
Household Food Insecurity	Secure	1.66(1.16 2.38)	.006
Cow Ownership	Yes	1.86(1.28, 2.70)	.001
Donkey Ownership	Yes	1.83(1.08, 3.11)	.024
Chicken Ownership	Yes	1.53(1.05, 2.22)	.027
Educational Status of the Mothers	Grades 5-8	1.74 (1.06, 2.86)	.028
	Grades 9 or above	2.96 (1.62, 5.42)	.000
Household Estimated Annual Income in Ethiopian Birr	20001-30000	2.22 (1.16, 4.26)	.017
**Flesh/meat Foods**			
Household Food Insecurity	Secure	5.08 (1.09, 23.71)	.039
**Eggs**			
Household food Insecurity	Secure	2.15 (1.33, 3.49)	.002
Root Crops Production	Yes	1.67 (1.05, 2.66)	.031
Chicken Ownership	Yes	3.20 (1.97, 5.19)	.000
**Overall ASF**			
Household Food Insecurity	Secure	1.94 (1.36, 2.78)	.000
Cow Ownership	Yes	1.89 (1.31, 2.74)	.001
Chicken Ownership	Yes	1.93 (1.33, 2.81)	.001
Estimated Household Annual Income	10001 – 20000	1.70 (1.06, 2.73)	.028
in Ethiopian Birr	20001 – 30000	1.96 (1.01, 3.79)	.047
	>30000	2.71 (1.36, 5.39)	.005
Educational Status of the Mothers	Grades 5-8	2.03 (1.25, 3.31)	.004

Discussion

All children are in need of care including nutrition that has positively to impact their healthy growth and development. They are recommended to be fed with diversified diets that include ASF [7,16]. Animal source foods are beneficial for the growth and development of infant and young childre[illegible]8,19] as they are important sources of complete protein and a range of micronutrient[[illegible]4]. In connection to this, the current study focused on the ASF consumption practice and its contributors among 6-23 months old IYC from selected rural districts in Ethiopia. To the result of this research, less than half (48.3%) of the children consu[illegible] at least one of the ASF (dairy or eggs or flesh foods). Dairy (41.2%) was better consume[illegible]an eggs (16.4%) and flesh foods (2.3%). The proportion of IYC who consumed flesh is comparable with reports from Aleta Wondo-3.4% [25], Gorche-2.6% [12], Dangila-2.4% [11], Wollo-3.8% [26] districts in Ethiopia, but lower than proportions from Addis Ababa city-15.3% [27], Afar-29.3% [28], Bale-8.9% [29] and Wolaita Sodo-17.7% [30] towns in Ethiopia. The observed difference might be subjected to study setting differences as stu[illegible] with higher proportion are conducted in city/towns where ASF consumption enabling factors are better [31]. Eggs are consumed by lower proportion of IYC than study done in Wollo-6.6% [26], Dejen-98% [32] and Afar-10.2% [28] districts in Ethiopia. It was also consumed by a lesser proportion of IYC than children studied by studies from Woliata Sodo-31.6% [30], Aleta Wondo-21.5% [25], Addis Ababa-29.8% [27] and Bale-42% [29]. Research conducted in Gorche-11% [12], Sinan-11% [33] and Dangila-11.7% [11] districts of Ethiopia reported almost equal proportion of children who consumed eggs compared with proportion found by this study. Overall, the results indicated that diets of considerable proportion of children are lacking animal source foods. Consequently, animal source food centric IYC nutrition activities are highly demanding in the study areas to improve their inclusion in the diets of children.

Household food security constantly increased probability of dairy, flesh and eggs consumption. The association could be explained by the inability [illegible] food insecure households' purchasing power with the increasing cost of ASF [34], and the fact that food insecure households prefer to consume less expensive food products to cope with food insecurity [35]. The odds of dairy consumption practice were increased by cow, donkey and chicken ownership. The positive contribution of cow ownership can be connected with being a direct source of the food item. As local market is the usual source of dairy products for majority of the households (63%), donkey's role in income generation might contributed to the positive association [36,37]. Chickens might have also contributed to the consumption of dairy in a similar income generation pathway [38] in addition of being direct source of eggs and possibly flesh foods. Similarly, odds of eggs consumption was more [illegible] children from households that ow[illegible] chick[illegible] Maternal better educational achievement was also associated with more likelihoo[illegible] dairy consumption. The overall ASF consumption analysis also showed a positive effect of mothers' higher educational achievement, cow or chicken ownership, better income category and household food security. The finding of a study done in Amhara-Ethiopia is in line with the

observed association between cow or chicken ownership and overall ASF consumpti[illegible]n the present study [39]. Thus, in addition to the need to reduce burden of food insecurity [illegible]hove for inclusion of animal source foods in diets of children demands consideration of improving livestock husbandry, strengtheni[illegible] nutrition sensitive income pathway and maternal empowerment [illegible]utrition l[illegible]cy. [illegible]ther usage and application of findings of the study shall be inconsideration of cur[illegible]f the study in identifying the study districts, selection of enumeration areas/kebeles and portion of participant drown from the enumeration areas. In addition, the study used single-day dietary recall method to assess animal source food consumption practice.

Conclusions

Majority of the IYC did not consume dairy products, eggs, flesh foods. Household food security was positively associated with all types of ASF and overall ASF consumption. Livestock (cow, chicken and donkey) ownership, better maternal edu[illegible]onal achievement and better household income increased IYC's dairy consumption chance. Root crops production and chick[illegible] ownership were positively related to eggs consumption. Overall ASF consumption was predicted by livestock ownership, household annual income and maternal education. In summary, consumption of ASF was dependent on socio-economic status and women empowerment [illegible] was measured by household food security, livestock ownership, household income and maternal educational status. Agricultural extension activities to increase healthy productivity aided by nutrition education should be considered and evaluated for their effect o[illegible]e IYC's ASF consumption practice in particular and diets in general. Women empowerment though education and income generation activities may also positively impact nutrition of IYC.

Acknowledgments

The authors acknowledge the School of Nutrition, Food Science and Technology-Hawassa University, Livestock System Intensification Lab-Project and administrative bodies of the study districts for the assistance they provided throughout the study process.

References

[1] Barker DJP. Developmental origins of chronic disease. Public Health 2012;126:185–9. https://doi.org/10.1016/j.puhe.2011.11.014.

[2] Benton D. The influence of children's diet on their cognition and behavior. Eur J Nutr 2008;47:25–37. https://doi.org/10.1007/s00394-008-3003-x.

[3] Martorell R. Improved Nutrition in the first 1000 Days and Adult Human Capita and Health. Physiol Behav 2017;29. https://doi.org/10.1016/j.physbeh.2017.03.040.

[4] Victora CG, De Onis M, Hallal PC, Blössner M, Shrimpton R. Worldwide timing of growth faltering: Revisiting implications for interventions. Pediatrics 2010;125. https://doi.org/10.1542/peds.2009-1519.

[5] Ethiopia M of HFDR of. Infant & Young Child Feeding 2010.

[6] Marriott BP, White A, Hadden L, Davies JC, Wallingford JC. World Health Organization (WHO) infant and young child feeding indicators: Associations with growth measures in 14 low-income countries. Matern Child Nutr 2012;8:354–70. https://doi.org/10.1111/j.1740-8709.2011.00380.x.

[7] World Health Organization. Essential Nutrition Actions: Improving Maternal, Newborn, Infant and Young Child Health and Nutrition 2013.

[8] Ministry of Health-Federal Democratic Republic of Ethiopia. Training of Trainers Manual for Counseling on Maternal , Infant and Young Child Nutrition 2011.

[9] Moursi MM, Arimond M, Dewey KG, Tre S, Ruel MT, Delpeuch F. Dietary Diversity Is a Good Predictor of the Micronutrient Density of the Diet of 6- to. J Nutr 2008:0–5. https://doi.org/10.3945/jn.108.093971.promise.

[10] Aemro M, Mesele M, Birhanu Z, Atenafu A. Dietary diversity and meal frequency practices among infant and young children aged 6-23 months in Ethiopia: A secondary analysis of Ethiopian Demographic and Health Survey 2011. J Nutr Metab 2013;2013:1–8. https://doi.org/10.1155/2013/782931.

[11] Beyene M, Worku AG, Wassie MM. Dietary diversity, meal frequency and associated factors among infant and young children in Northwest Ethiopia: A cross-sectional study. BMC Public Health 2015;15. https://doi.org/10.1186/s12889-015-2333-x.

[12] Dangura D, Gebremedhin S. Dietary diversity and associated factors among children 6-23 months of age in Gorche district, Southern Ethiopia: Cross-sectional study. BMC Pediatr 2017;17:1–7. https://doi.org/10.1186/s12887-016-0764-x.

[13] Forsido SF, Kiyak N, Belachew T, Hensel O. Complementary feeding practices, dietary diversity,

and nutrient composition of complementary foods of children 6-24 months old in Jimma Zone, Southwest Ethiopia. J Health Popul Nutr 2019;38:14. https://doi.org/10.1186/s41043-019-0172-6.

[14] Lemeshow S, Jr DWH, Klar J, Lwanga SK. Adequacy of Sample Size in Health Studies. Biometrics 1991;47:347. https://doi.org/10.2307/2532527.

[15] Coates, J., Swindale, A. and Bilinsky P. Household Food Insecurity Access Scale (HFIAS) for Measurement of Household Food Access: Indicator Guide Version 3. Food and Nutrition Technical Assistance Project Academy for Educational Development, Washington DC. Acad Educ Dev 2013;53:1689–99. https://doi.org/10.1017/CBO9781107415324.004.

[16] World Health Organization and the United Nations Children's Fund (UNICEF). Indicators for assessing infant and young child feeding practices. World Heal Organ United Nations Child Fund 2008;WHA55 A55/:19.

[17] Bendel RB, Afifi AA. Comparison of stopping rules in forward "stepwise" regression. J Am Stat Assoc 1977;72:46–53. https://doi.org/10.1080/01621459.1977.10479905.

[18] Headey D, Hirvonen K, Hoddinott J. Animal sourced foods and child stunting. Am J Agric Econ 2018;100:1302–19. https://doi.org/10.1093/ajae/aay053.

[19] Herforth A, Arimond M, Álvarez-Sánchez C, Coates J, Christianson K, Muehlhoff E. A Global Review of Food-Based Dietary Guidelines. Adv Nutr 2019;10:590–605. https://doi.org/10.1093/advances/nmy130.

[20] Murphy SP, Allen LH. Nutritional Importance of Animal Source Foods. J Nutr 2003;133:3965–71.

[21] Whaley SE, Sigman M, Neumann C, Bwibo N, Guthrie D, Weiss RE, et al. Animal Source Foods to Improve Micronutrient Nutrition and Human Function in Developing Countries. J Nutr 2003;133:3965–71.

[22] Zhang Z, Goldsmith PD, Winter-Nelson A. The Importance of Animal Source Foods for Nutrient Sufficiency in the Developing World: The Zambia Scenario. Food Nutr Bull 2016;37:303–16. https://doi.org/10.1177/0379572116647823.

[23] Neumann C, Harris DM, Rogers LM. Contribution of animal source foods in improving diet quality and function in children in the developing world. Nutr Res 2002;22:193–220. https://doi.org/10.1016/S0271-5317(01)00374-8.

[24] Whaley SE, Sigman M, Neumann C, Bwibo N, Guthrie D, Weiss RE, et al. Providing Micronutrients through Food-Based Solutions: A Key to Human and National Development. J Nutr 2003;133:3965–71.

[25] Dafursa K, Gebremedhin S. Dietary Diversity among Children Aged 6-23 Months in Aleta Wondo District, Southern Ethiopia. J Nutr Metab 2019;2019. https://doi.org/10.1155/2019/2869424.

[26] Gebremedhin S, Baye K, Bekele T, Tharaney M, Asrat Y, Abebe Y, et al. Predictors of dietary diversity in children ages 6 to 23 mo in largely food-insecure area of South Wollo, Ethiopia. Nutrition 2017;33:163–8. https://doi.org/10.1016/j.nut.2016.06.002.

[27] Solomon D, Aderaw Z, Tegegne TK. Minimum dietary diversity and associated factors among children aged 6-23 months in Addis Ababa, Ethiopia. Int J Equity Health 2017;16:1–9. https://doi.org/10.1186/s12939-017-0680-1.

[28] Wuneh AG, Ahmed W, Bezabih AM, Reddy PS. Dietary Diversity and Meal Frequency Practices among Children Aged 6-23 Months in Agro Pastoral Communities in Afar Region, Ethiopia: A Cross-sectional Study. Ecol Food Nutr 2019;58:575–96. https://doi.org/10.1080/03670244.2019.1644328.

[29] Tegegne M, Sileshi S, Benti T, Teshome M, Woldie H. Factors associated with minimal meal frequency and dietary diversity practices among infants and young children in the predominantly agrarian society of Bale zone, Southeast Ethiopia: A community based cross sectional study. Arch Public Heal 2017;75:1–11. https://doi.org/10.1186/s13690-017-0216-6.

[30] Mekonnen TC, Workie SB, Yimer TM, Mersha WF. Meal frequency and dietary diversity feeding practices among children 6-23 months of age in Wolaita Sodo town, Southern Ethiopia. J Health Popul Nutr 2017;36:18. https://doi.org/10.1186/s41043-017-0097-x.

[31] Streeter JL. Socioeconomic Factors Affecting Food Consumption and Nutrition in China: Empirical Evidence During the 1989–2009 Period. Chinese Econ 2017;50:168–92. https://doi.org/10.1080/10971475.2017.1297653.

[32] Kumera G, Tsedal E, Ayana M. Dietary diversity and associated factors among children of Orthodox Christian mothers/caregivers during the fasting season in Dejen District, North West Ethiopia. Nutr Metab 2018;15:1–9. https://doi.org/10.1186/s12986-018-0248-0.

[33] Temesgen H, Yeneabat T, Teshome M. Dietary diversity and associated factors among children aged 6–23 months in Sinan Woreda, Northwest Ethiopia: a cross-sectional study. BMC Nutr 2018;4:1–8. https://doi.org/10.1186/s40795-018-0214-2.

[34] Minten B, Fantu B. Improving the evidence and policies for better performing livestock systems in Ethiopia. 2020.

[35] Asesefa Kisi M, Tamiru D, Teshome MS, Tamiru M, Feyissa GT. Household food insecurity and coping strategies among pensioners in Jimma Town, South West Ethiopia. BMC Public Health 2018;18:1–8. https://doi.org/10.1186/s12889-018-6291-y.

[36] Angara, T. E. E., Ismail, A. A. and Ibrahim AM. THE ROLE OF DONKEYS IN INCOME GENERATION AND THE IMPACT OF ENDOPARASITES ON THEIR PERFORMANCE. J Veternary Med Anim Prod 2011;2:65–89.

[37] Valette D. Invisible Workers. The Economic Contributions of Working Donkeys, Horses and Mules to Livelihoods. 2015. https://doi.org/10.1177/001139287035003004.

[38] Hundie D, Goshu G, Tamir B, Duguma G. Assessment on rural poultry production and marketing system of Horro chicken ecotypes in Western Ethiopia 2019;11:248–59. https://doi.org/10.5897/JAERD2019.1046.

[39] Kim SS, Nguyen PH, Tran LM, Abebe Y, Asrat Y, Tharaney M, et al. Maternal behavioural determinants and livestock ownership are associated with animal source food consumption among young children during fasting in rural Ethiopia. Matern Child Nutr 2019;15:1–9. https://doi.org/10.1111/mcn.12695

Supporting information

S1 Table. Table 4 Bivariate logistic regression on dairy consumption of infant and young children.

S2 Table. Table 5 Bivariate logistic regression analysis on Flesh/Meat Consumption of infant and young children.

S3 Table. Table 6 Bivariate logistic regression analysis on eggs consumption of infant and young children.

S4 Table. Table 7 Bivariate logistic regression on overall ASF consumption of infant and young children.
