# Supplementary material for: Animal source food consumption practice and factors associated among infant and young children from selected rural districts in Ethiopia: A cross-sectional study
Source: PLoS One. 2024 Jul 5;19(7):e0306648. doi: 10.1371/journal.pone.0306648 (PMC11226061; doi:10.1371/journal.pone.0306648)
Supplement: Attachment — Submitted filename: Response to Reviewer.pdf [file pone.0306648.s009.pdf]

November 28, 2023

Subject: <u>Point-by-point response to Editors guidance and reviewer's comments</u>

Dear Editor,

Thank you for giving us the opportunity to submit a revised draft of the manuscript "Animal source food consumption practice and factors associated among infant and young children from selected rural districts in Ethiopia: A cross-sectional study" for publication in PLOSOne. We would like to thank the reviewer for her/his precious time and constructive comments. We have addressed the comments in a point- by- point response and a revised manuscript. The changes are highlighted with track changes in the revised manuscript.

Point-by-Point Responses;

- Need for grammatical corrections throughout the manuscript
  - ✓ Grammatical errors are addressed following recommendation form the reviewer and with the help of language expert. We would love also to thank the reviewer for making recommendation in the manuscript.
- Abstract: Provide prevalence of ASF consumption
  - ✓ Results are included in the results subsection of the abstract.
- The need to update criteria for IYCF assessment
  - ✓ Concepts and references are updated throughout the manuscript
- Additional citations are needed
  - ✓ More sources are cited in the manuscript including sources recommended by the reviewer. THANKS!
- Data Collection section of the manuscript the reviewer requested the authors to articulate on how the recall bias was assessed for the sentence "*Household food in/security was assessed on a recall basis during thirty days before the date of data collection*."
  - ✓ We kindly request the reviewer to capture that the sentence is not about recall bias assessment. It is about how household food insecurity was assessed. This is to mean respondents we requested to recall their household-level food insecurity related experience in the past month, 30 days before the day of data collection.

- Have the authors considered adjusting for confounders in the multivariable regression?
    - ✓ Yes! The analysis was done two times (first bivariate and secondly multivariable) for each of the ASF and overall ASF consumption practice. Multivariable regression is one of the approaches with which we control for possible confounder during analysis. Results of the bivariate analysis are provided as a Supplementary Tables and variables considered in the multivariable regression model are listed as a foot note.
- Seemingly, determinants in the multivariate models could be correlated. I see the authors have investigated this, but it would be valuable for the authors to present their check on multicollinearity based on VIF.
    - ✓ Yes, correlation between independent variables was assessed using variance inflation factor and it is described in the manuscript. Now, we have provided VIF related information on the foot note.
- Discussion section: The reviewer requested to further discuss the discussion we provided about the association between Donkey or Chicken ownership and dairy products consumption practice.
    - ✓ Thanks for these value adding views so that our narrations are to the level of message we wanted to convey. Additional sentences are included to support readers understanding and avoid misinterpretation.

With Best regards,

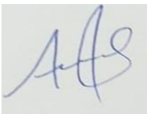

Alemneh Kabeta Daba (PhD)
