# Supplementary material for: Animal source food consumption practice and factors associated among infant and young children from selected rural districts in Ethiopia: A cross-sectional study
Source: PLoS One. 2024 Jul 5;19(7):e0306648. doi: 10.1371/journal.pone.0306648 (PMC11226061; doi:10.1371/journal.pone.0306648)
Supplement: Attachment — Submitted filename: Response to Reviewer.pdf [file pone.0306648.s010.pdf]

April 15, 2024

Subject: <u>Point-by-point response to Editors guidance and reviewer's comments</u>

Dear Editor,

Thank you for giving us the opportunity to submit a revised version of the manuscript with title of "***Animal source food consumption practice and factors associated among infant and young children from selected rural districts in Ethiopia: A cross-sectional study***" for publication in PLOSOne. We would like to thank the reviewer for her/his precious time, constructive comments, teaching views and diligence for the betterment of our research output. We have addressed the comment in a point- by- point response below. This round we are not submitting revised manuscript with track changes as we only justified why we shall not revise the manuscript according to reviewer's view.

Point-by-Point Response;

- Reviewer #3: While the authors have addressed many comments from the past iteration, it seems the results of regression analysis involving MDD have not been updated after the new cutoff is implemented - all the regression results are still the same as the past version. This needs to be addressed.
  - ✓ Dear Dehao Chen, thanks for your time, contribution, teaching views and diligence for the betterment of our research output. Let me/us use this as an opportunity that we enjoyed your comments and learnt a lot from your 360° views. Coming to the insightful comment provided….Yes, as the study period was May to Augest 2018, we used the revised (cited as [27]) standard dietary diversity assessment questionnaire for children under-two. However, from the data collected (eight food groups including breast milk) using the standard child dietary diversity assessment questionnaire, our being processed manuscript focused on selected animal source foods (Dairy products, Eggs and Flesh foods) consumption practice of children 6-24 months as outcome variables [***Results Section-Dairy products, eggs, flesh foods and overall ASF consumption practice of the IYC section of the manuscript in the results sub-section***], not MDD. Also,

The logistic regression analyses were done to identify factors associated with IYC's consumption practice of each of the selected animal source food and overall animal source food [***Results-Section-Factors associated with animal source foods consumption practice & Table 3***], not factors associated with MDD. To this end, though we revised the citation for source document of the data collection questionnaire we used for the outcome variables, we do not believe that the results from the regression analyses has to be updated.

KIndly,

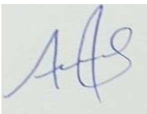

Alemneh Kabeta Daba (PhD)
