# Supplementary material for: Animal source food consumption practice and factors associated among infant and young children from selected rural districts in Ethiopia: A cross-sectional study
Source: PLoS One. 2024 Jul 5;19(7):e0306648. doi: 10.1371/journal.pone.0306648 (PMC11226061; doi:10.1371/journal.pone.0306648)
Supplement: Attachment — Submitted filename: Reviewer Report.docx [file pone.0306648.s006.docx]

**Manuscript title**: Predictors of Animal Source Food Consumption among Infant and Young Children from Rural Districts of Ethiopia: A Cross-sectional Study

Manuscript Number: PONE-D-22-30710

**Reviewer Report on the submitted manuscript**

I. **General Comment**

It is an important research topic but the quality of the reporting and validity and reproducibility of methods employed to address the stated problem is unsatisfactory. The results and discussion sections need more improvement. Moreover, grammatical and editorial errors are frequent thought the document and thus the manuscript needs major revision.

**II. Specific Comments**

**Title:** The title needs modification in light of the cross-sectional nature of the study design, authors can’t determine predictors of animal Sources Food Consumption among Infants and Young Children using a cross-sectional study design. For determining predictors authors need to use either a cohort or an interventional study design. By using a cross-sectional study, one can determine only the associated factors for the outcome variable. Moreover, the phrase “Rural Districts of Ethiopia” is very broad considering the large surface area of Ethiopia.

**Abstract:**

- In the background section of the abstract, the problem statement and rationale for conducting the study are missing. There is a miss-match between the title and the specific objectives described in the abstract. In the title, “Determination of predictors …” while in the objective “assess consumption of ASF and identify their predictors”.
- In the method section: Authors failed to describe the specific study area, the sampling procedure, the type of data collected, who collected the data, and what type of descriptive analysis was carried out.
- Results Section: the authors used logistic regression analysis to identify associated factors with animal source food consumption. However, they use p values to report results. They should use AOR along with 95% CI as this help us to report the statistical significance, strength of association and direction of association.

**Introduction:** Authors need to explicitly describe the problem statement for their study.

**Methods:**

- The study areas were not adequately described including population distribution, geography, socio-economy status and health infrastructure etc.
- On Page 4, 2^nd^ paragraph, the authors used “Sampling” as a title for Sample Size Determination and Sampling Procedure. The word sampling is incomplete and thus should be replaced by Sample Size Determination and Sampling Procedure.
- In sample size calculation, authors didn’t provide the expected prevalence assumed for calculation of the sample size and didn’t consider for non-response.
- In the sampling procedure, authors said that they selected randomly four Kebelle from each district but they didn’t elaborate why they selected four Kebelles as number of Kebelles can vary from district to district. They didn’t provide also justification why they selected 25 study subjects from each Kebelle. There is no “Equal Proportional Allocation” sampling technique. It is not also clear how the systematic sampling method was conducted.
- On Page 4, 3^rd^ paragraph, First sentence, Authors wrote that “*Data were collected through one-on-one interviewer administered questionnaire*”. What does “One-on-one” mean? Is it meant to say face-to-face?
- Authors didn’t describe how they measured the independent and dependent variables. No operational definition was given. No information was given as to who collected the data.
- The data quality control used was inadequate. Did authors translate their tool to local language? Did they use supervision during data collection? Did they check completeness of the collected data on daily basis?
- With regards to ethical consideration, authors claimed that “*All methods were carried out in accordance with the Declaration of Helsinki*”. This is vague, incomplete and inappropriate description. This should not be described under the “Data collection” heading. There is no information as to which University Institutional Review Board gave the permission? Dis authors use verbal or written informed consent? What did they do to ensure autonomy, beneficence and do not harm?
- In the data analysis section, authors failed to include multicollinearity test and goodness of fit test for the final logistic model.

**Results:**

- What is the response rate for the study? It needs to be stated.
- On page 6, Table 1: Second column should be labeled as categories; in Table 1 and Table 2, the column label for the third and fourth column should be revised as Frequency (N) and Percentage (%), respectively. What is “Age completed years” mean in Table 1? It is not clear and why it is categorized as ≤ 26 and ≥ 27.
- On Page 8, Table 2: The reason for categorizing family size, crop production and livestock ownership is not clear. Family size is categorized into ≤5 and ≤6. In the crop production and livestock ownership, what if a farmer produces two, three, four, five or all type of crops and a farmer owns two or more of the livestock?
- On Page 9: The y-axis of figure is not labeled
- Page 11, Table 3: The outcome variable (animal source food consumption) frequency and percentage is missing in Table 3. It doesn’t include also the bivariate analysis results (COR along with the 95% CI). Why is donkey ownership included in Table 3 while Table is about animal source food consumption? How ownership of donkey is can be associated with the consumption of animal source foods? The bivariate and multivariable results should be presented in the same Table. It is not also clear why some variables and categories are missed in Table 3.

**Discussion:**

- Generally the discussion section is very brief considering the study objectives and the findings.
- On page 12, 1^st^ paragraph, comparison of proportion from various areas was based narrative descriptions. It needs to be supported by data/figures. For example in the sentence “*The proportion of IYC who consumed flesh is comparable with reports from Aleta Wondo [25], Gorche [12], Dangila [11], Wollo [26] districts in Ethiopia, but lower than proportions from Addis Ababa city [27], Afar [28], Bale [29] and Wolaita Sodo [30] towns in Ethiopia*” what was the figure for Aleta Wondo? Gorche? Dangila? Wollo? etc. to say greater than or lower than the current study. Moreover, implications of the findings to clinical/public health practices need to be described.
- On page 12, 2nd paragraph, associated factors (“Predictors”) with the consumption of animal source food are discussed. However, the results are not compared with other findings. Simiarly, implications of the findings are not discussed.
- Limitations of the study related to selection and measurement biases and confounding are not discussed.
